# Case report: Double filtration plasmapheresis (DFPP) for severe rhesus-D alloimmunization in two pregnant patients

**DOI:** 10.3389/fped.2023.1147675

**Published:** 2023-04-11

**Authors:** Yuling Liang, Tenghui Wang, Wenjian Zhu, Xiaohua Wang, Xuemei Zhang, Zhihua Zheng, Yan Lei

**Affiliations:** Department of Nephrology, Center of Nephrology and Urology, The Seventh Affiliated Hospital, Sun Yat-sen University, Shenzhen, China

**Keywords:** double filtration plasmapheresis, erythrocyte alloimmunization, intrauterine blood transfusion, anti-D antibodies, pregnancy

## Abstract

Maternal erythrocyte alloimmunization is one of the most important causes of fetal anemia. The standard treatment for anemic fetuses is intrauterine blood transfusion (IUT). However, IUT may have adverse effects, particularly before 20 weeks of gestation. In this report, two women who had previously had severely affected alloimmunized pregnancy developed high titers of anti-D antibodies before 20 weeks of gestation. Ultrasound Doppler showed severe fetal anemia, and intrauterine transfusion was expected to be unavoidable. To prolong pregnancy to a gestation in which intravascular IUT was possible, we used repeated double filtration plasmapheresis (DFPP) as a rescue therapy. The titers of IgG-D, IgG-A, and IgG-B decreased after DFPP treatment. One woman successfully prolonged pregnancy until 20 weeks of gestation. Subsequently, she underwent four cycles of IUTs and delivered at 30 weeks of gestation by emergency cesarean section due to fetal bradycardia during the fifth intrauterine transfusion. The other woman successfully delayed intrauterine transfusion until 26 weeks of gestation. The favorable results of the two patients indicate that DFPP may be an effective and safe treatment modality for RhD immunity in pregnant women. Moreover, DFPP is potentially helpful for reducing the occurrence of ABO hemolytic disease in neonates due to the clearance of IgG-A and IgG-B antibodies (e.g., O pregnant women harbored A/B/AB neonates). However, more clinical trials are needed to verify the results.

## Introduction

Rhesus incompatibility during pregnancy is a maternal-fetal erythrocyte antigen mismatch, belonging to the rhesus blood group system (most commonly D, E, e, C, and c). Rh(D) alloimmunization leading to hemolytic disease of the fetus and neonate remains an important cause of perinatal mortality, morbidity, and long-term disability. When an RhD-negative mother is pregnant with an RhD-positive fetus, she may be exposed to RhD-positive red blood cells (RBCs) from the fetus during an abortion, a delivery, an amniocentesis, and a blood transfusion, and be sensitized to produce anti-D. This antibody can enter the fetal blood circulation through the placenta and bind to the RhD antigen on the fetal RBCs, leading to fetal and neonatal hemolytic disease, which can cause neurological disorders and even death in severe cases (1–3).

Starting in the late 1960s, the administration of anti-D immunoglobulin (RhIg) to RhD-negative women immediately after delivery has greatly reduced the morbidity and mortality of the disease, as well as its severity (3–7). Although immunoprophylaxis achieved good results, cases of RhD-related fetal hemolysis still occur. It has been reported that the introduction of postpartum RhIg in the late 1960s has resulted in only a 50% decrease in RhD disease globally (6). The reasons why hemolytic diseases in fetuses and neonates still occur include: inadequate prenatal care due to a lack of awareness and financial constraints; lack of preventive measures (especially for women from countries with low levels of health care); the anti-D immunoprophylaxis dose is too small for effective prevention; and the mother was sensitized by a blood transfusion (4).

The first-line treatment for anemic fetuses is intrauterine blood transfusion (IUT). However, early IUT before 20 weeks is more technically challenging, resulting in a higher risk of complications, such as intrauterine infection, premature rupture of membranes, emergency delivery, and even fetal death (8, 9). Currently, there are no guidelines based on reliable data for uniform therapeutic intervention in cases of severe early-onset RhD alloimmunization before 20 weeks of gestation. It has been reported that intravenous RhIg before 20 weeks of gestation may delay early intrauterine transfusions in women at risk of maternal-fetal hemolysis compared with those who did not use intravenous RhIg (10).

Therapeutic plasma exchange (TPE), a technique based on blood plasma removal and replacement, is recommended with a low level of evidence, and as such is classified as a category 3 grade 2C therapeutic apheresis by the American Society of Apheresis (ASFA). In other non-pregnant populations, DFPP, a technique based on plasma filtration, has been reported to be effective (11). It was reported that DFPP is a safe and well-tolerated apheresis method, with less or no replacement of blood products than TPE (12). However, the use of DFPP in the treatment of alloimmune pregnancy is rare and mostly based on case reports, only two of which referred to RhD alloimmunity (13–16). Bek SG el at. reported that RhD alloimmunization was treated by DFPP, but the procedure was performed after 20 weeks of gestation (15). DFPP for RhD incompatible pregnancy has been performed between 15 and 35 weeks of gestation (16). Herein, we report two cases of RhD-resensitized pregnant women who underwent repeated DFPP before 20 weeks of gestation, which successfully delayed IUT until after 20 weeks of gestation, at which point IUT could be safely carried out, leading to successful pregnancies with live born infants.

## Case reports

### Case 1

**Figure 1:**
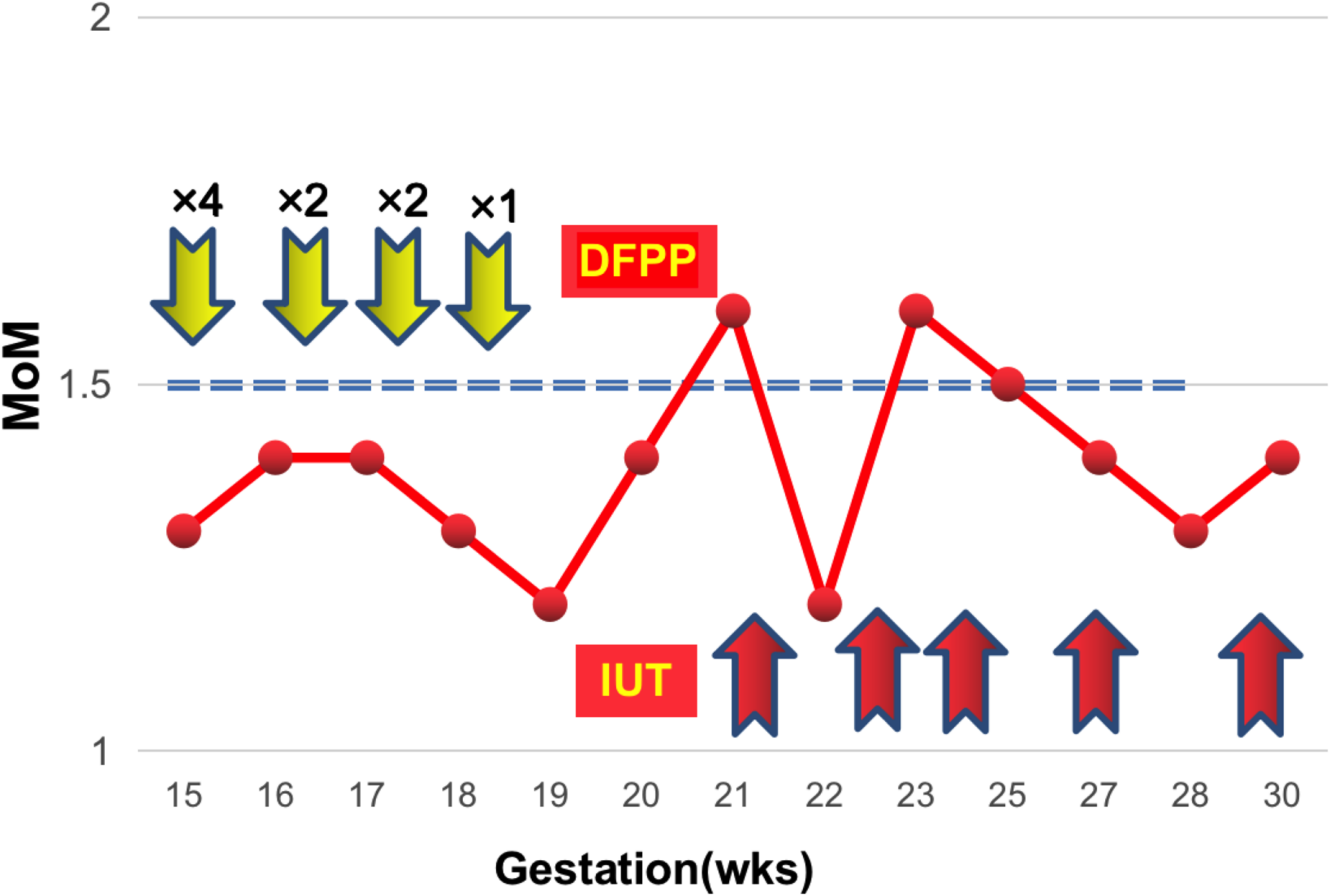
The fluctuations of MoM during the whole treatment course (case 1). The yellow arrows denote the DFPP treatment session. The red arrows indicate IUT.

**Figure 2:**
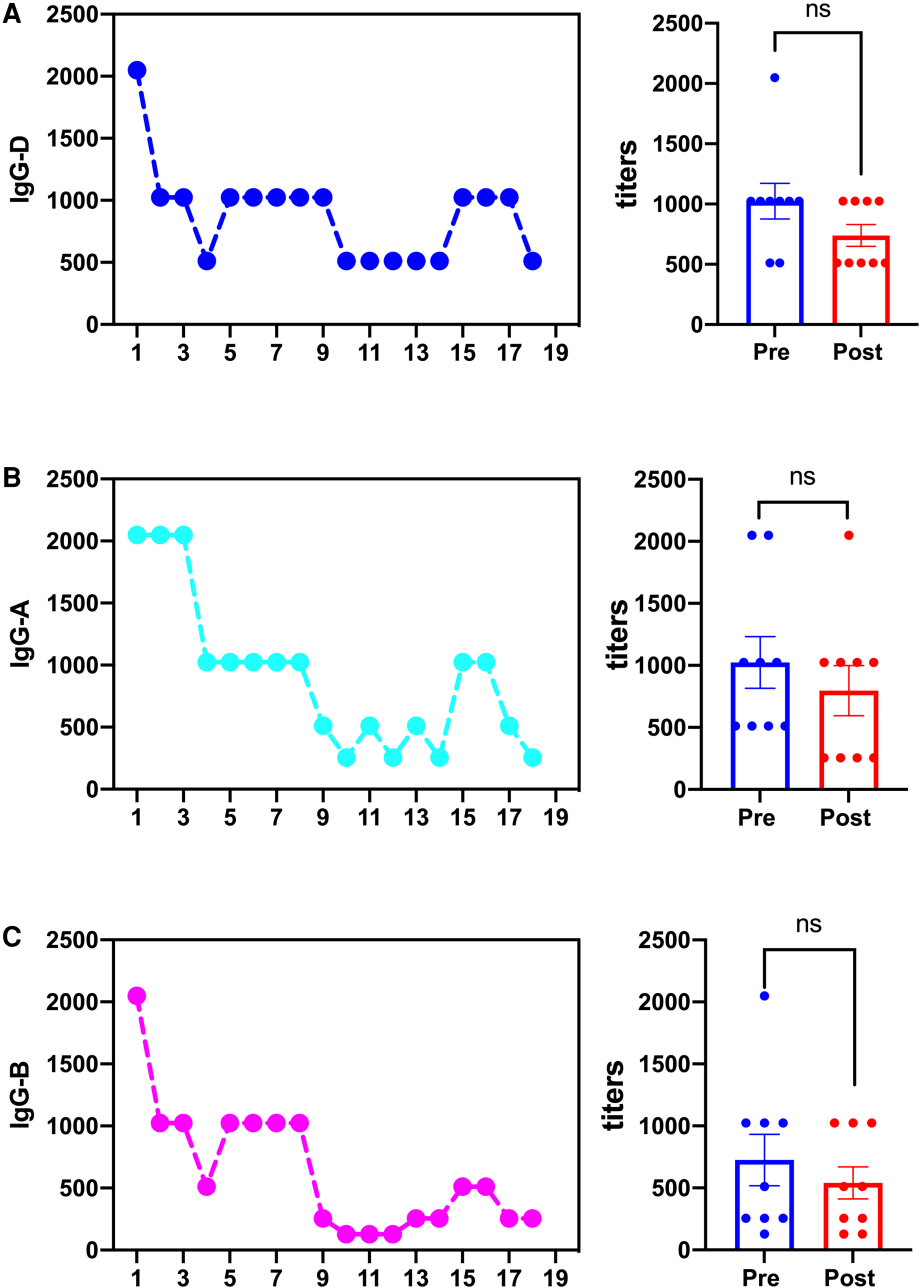
The changes of IgG-D, (**A**), and (**B**) antibodies during DFPP treatment (case 1). (**A**) The change of IgG-D titers and the comparison of IgG-D titers pre- and post- DFPP treatment. (**B**) The change of IgG-A titers and the comparison of IgG-A titers pre- and post- DFPP treatment. (**C**) The change of IgG-B titers and the comparison of IgG-B titers pre- and post- DFPP treatment. odd numbers represent pre-DFPP. ns, not significant.

**Figure 3:**
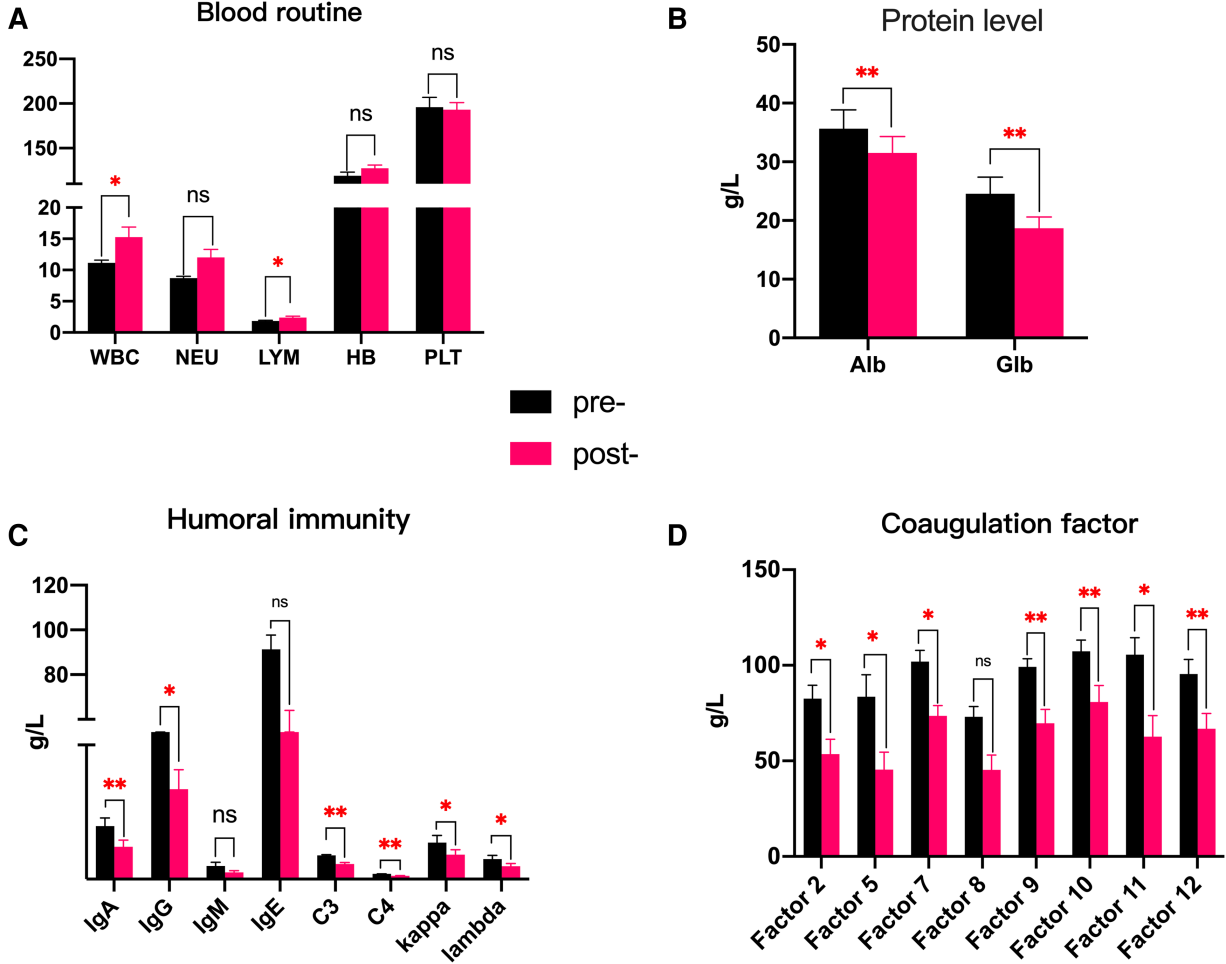
(**A–D**) Comparison of the changes in blood routine and protein levels pre- and post-DFPP treatment (case 1). *p < 0.05; **p < 0.01; ns, not significant.

**Figure 4:**
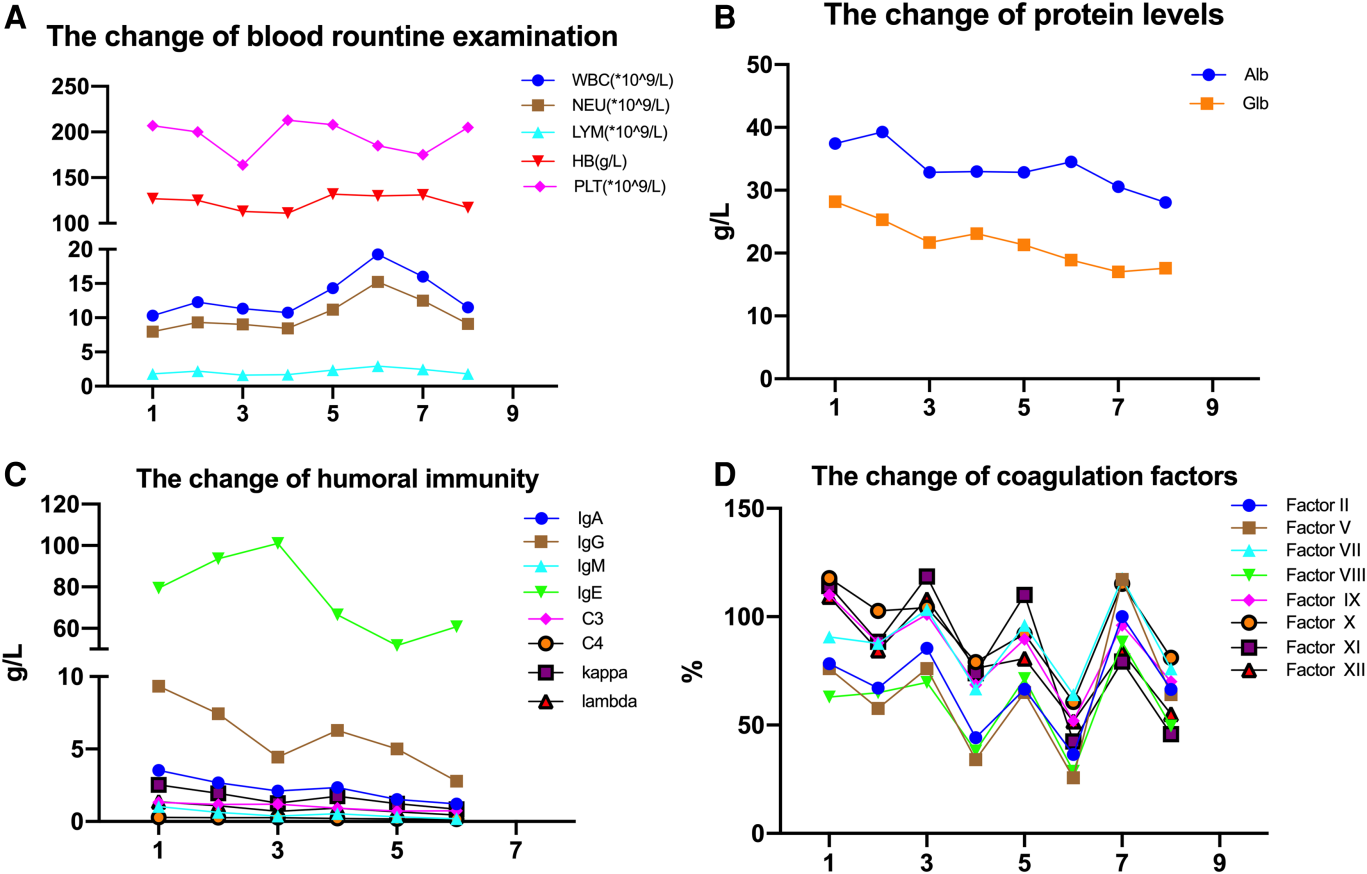
(**A–D**) the fluctuations of blood routine and protein level over the whole treatment period (case 1). Odd numbers represent pre-DFPP. Reference range: WBC, 3.5–9.5*10^9^/L; NEU, 1.8–6.3*10^9^/L; LYM, 1.1–3.2*10^9^/L; Hb, 115–150 g/L; Alb, 40–55 g/L; Glb, 20–40 g/L; IgA, 0.7–4.0 g/L; IgG, 7–16 g/L; IgM, 0.4–2.3 g/L; C3, 0.9–1.8 g/L; C4, 0.1–0.4 g/L; kappa, 1.7–3.7 g/L; lambda, 0.9–2.1 g/L; factor II, 70–100 (%); factor V, 70–120 (%); factor VII, 70–120 (%); factor VIII, 70–150 (%); factor IX, 70–120 (%); factor X, 70–120 (%); factor XI, 70–120 (%); factor XII 70–150 (%).

This 27-year-old woman, whose blood group was O RhD negative, was in her third pregnancy. At the age of 24, she became pregnant with her first husband (AB, RhD positive) and gave birth to a healthy male infant by cesarean section. Within 72 h after delivery, she received two anti-D immunoglobulin injections. When she was 26 years old, she became pregnant with her second husband (AB, RhD positive). At 32 weeks of gestation, she first tested positive for anti-D and received two RhIg injections as a rescue therapy in a local hospital; the injections had no effect on this pregnancy. A male infant, whose blood group was B RhD positive), was delivered by cesarean section at 37 weeks of gestation. He died 5 h after birth due to severe RhD incompatible hemolytic anemia, heart failure, and severe asphyxia. In the present pregnancy, the anti-D antibody titer was up to 2,048 at 13 weeks of gestation. Fetal anemia was assessed by the peak velocity of systolic blood flow in the middle cerebral artery (MCA-PSV) detected by Ultrasound Doppler, which fluctuated between 1.3 and 1.5 multiples of the median (MoM). A total of 9 cycles of DFPP were administered between 14 and 20 weeks of gestation (Figure 1). The DFPP procedure was performed using a plasmapheresis machine (Multifiltrate CiCa ®, FMC, Bad Homburg, Germany), a plasma separator (P2, Fresenius, Germany), and a plasma component separator (Cascadeflo EC-30W, Asahi Kasei Medical Co, Japan). A volume of 600 ml of 5% albumin was used as replacement fluid. The ight jugular vein was intubated for vascular access during the treatment run. The blood flow rate was 100–120 ml/min and the plasma separation rate was 1,000 ml/h. Plasma volume was calculated using the Kaplan formula: plasma volume estimate = [0.065 * weight (kg)] * (1-hematocrit). Anticoagulation was achieved using low molecular weight heparin. Epidemiological data, hematological parameters, and side effects were evaluated before and after DFPP. After DFPP treatment, anti-D IgG antibody was removed from the serum, along with other IgG (anti-A and anti-B) (Figure 2) and IgM (anti-A and anti-B) antibodies (Supplementary Material 1). During the whole treatment period, the titers of anti-D stayed between 512 and 1,024 and MCA-PSV stayed below 1.5 MoM until 20 weeks of gestation before IUT. Additionally, other proteins, such as albumins, globulins, and coagulation factors, decreased after DFPP treatment, while blood routine did not (Figure 3). Globulins may be needed to replenish after DFPP treatment for serum level of globulin decreasing to lower level. However, coagulation factors rebounded to the normal level on the next day and albumin was still above 30 g/L (Figure 4); therefore, protein supplements were not needed. No obvious side effects were observed during the entire treatment period. From 21 to 27 weeks of gestation, MCA-PSV increased to 1.63 MoM, and four IUTs were performed successfully. Owing to bradycardia during the fifth IUT at 30 weeks of gestation, an emergency cesarean section was performed, and a female infant was delivered with an Apgar score of 1, 4, 4 points. Her hemoglobulin level was 90 g/L. Respiratory support and blood transfusion restored the infant's health.

### Case 2

This 38-year-old woman, whose blood group was AB RhD negative, was in her sixth pregnancy. She had self-induced abortion twice due to social factors when she was 26 and 27 years old and had spontaneous abortion twice due to embryo termination at the ages of 30 and 31. She had her fifth pregnancy at the age of 34 and anti-D antibody had a maximum titer of 1,024. Cesarean section was performed at 35 weeks of gestation due to severe fetal anemia. The peripheral blood of the newborn was type A RhD-positive with 68 g/L of hemoglobin and positive in a Coombs test. The newborn was diagnosed with RhD incompatibility hemolysis. After blood transfusion and exchange transfusion treatment, he is now healthy. For her sixth pregnancy, the anti-D antibody titer was 512 at 8 weeks of gestation. MCA-PSV increased to 1.51 MoM at 16 weeks of gestation. She received three cycles of DFPP between 18 and 19 weeks of gestation. Hypoalbuminemia (as low as 24.72 g/L) and hypofibrinogen (as low as 1.01 g/L) were observed, and 2 g of fibrinogen was administered intravenously to reduce the risk of bleeding. After DFPP treatment, MCA-PSV decreased to 1.32 MoM and the titer of anti-D antibody dropped to 256 (Supplementary Material 2). At 26 weeks of gestation, an ultrasound showed cardiac dilatation, with MCA-PSV increasing up to 1.55 MoM, and four cycles of IUT (Supplementary Material 3) were performed with no adverse reactions until 36 weeks of gestation. Owing to tachycardia and fever at 36 weeks of gestation, an emergency cesarean section was performed, and a female infant was delivered with an Apgar score of 10, 10, 10 points and a birth weight of 2.83 kg. Her initial tests revealed a hemoglobin level of 126 g/L and an A RhD-positive blood type. She was yellow stained after birth. After receiving respiratory support, whole blood exchange, phototherapy, and IVIG (2 g/kg), she was healthy. On postnatal day 6, the baby was discharged with a body weight of 2.925 kg.

## Discussion

The prevalence of RhD-negative women varies widely across the globe. Among white women, the rate is approximately 15%, whereas in Asia, including China, Japan, and Indonesia, it is only approximately 0.5% (17). Although the incidence of the RhD-negative blood group in Chinese women is low, the incidence of maternal-fetal RhD alloimmunization will increase with the implementation of the three-child policy in China.

All RhD-negative pregnant women are considered at risk for fetal anemia and need to be monitored. Since its introduction in 1981, IUT has become a cornerstone of treatment management for fetuses with severe anemia due to RhD incompatibility during pregnancy (18). However, IUT is not always feasible as it can lead to intrauterine infection, premature rupture of membranes, emergency delivery, and even fetal death, especially if performed before 20 weeks of gestation. Early-onset RhD alloimmunization in pregnancy, more commonly seen among women who have been alloimmunized previously, is not only associated with more severe fetal anemia, a higher risk of fetal hydrops, and intrauterine death, but is also more technically challenging to manage with standard IUT therapy. During early pregnancy, safe access to the fetus or umbilical cord vasculature is rarely possible (8–10). Several strategies have been investigated to delay the progression of severe fetal anemia and prolong pregnancy to the point at which IUT is safe, including RhIg and maternal plasma exchange and maternal DFPP (19).

The American Apheresis Society proposed that therapeutic plasma exchange should be considered early for fetal hemolytic anemia at 7–20 weeks of gestation until IUT can be safely performed as a first-line treatment after 20 weeks (11). The principle of plasma exchange is to remove antibodies against RBCs from the maternal circulation, thus decreasing the number of antibodies that can cross the placenta and destroy fetal erythrocytes; this is followed by replenishment with an equal amount of plasma. Although PE is a simple procedure, it is expensive for patients. In addition, it requires a large volume of donated plasm, which carries the risk of infection with known or unknown pathogens. More importantly, owing to the low incidence of the RhD-negative blood group in China, the supply of RhD-negative plasma is extremely scarce; therefore, it is difficult to meet the demand for PE. RhD-positive plasma has been used as replacement fluid in TPE treatment; although this is a generally accepted practice, there is a case report of a pregnant woman with repeated severe RhD-sensitization who was given RhD-positive fresh frozen plasma (FFP) as part of a plasma exchange, resulting in increased anti-RhD titers and delayed neonatal RhD-incompatible hemolysis (20). This may be related to anti-D alloimmunization caused by residual RhD-positive erythrocytes in fresh frozen plasma (FFP). Residual RBCs within FFP may cause erythrocyte alloimmunity, and identification of anti-D has been reported after plasma transfusions (21–23). To minimize the contamination of RBCs, the Council of Europe recommendation guide has set a limit of 6.0 × 10^9^/L RBCs in clinical FFP (24). There is no standard for the maximum concentration of RBCs in a plasma unit in China. The RhD status of the donor plasma should be considered as an alternative solution for plasma exchange from RhD-negative women.

DFPP processing is based on two filters with different pore sizes. Pathogenic substances (higher molecular weight proteins) are discarded from separated plasma using plasma filters with different pore sizes, which are mainly determined by the molecular weight and three-dimensional configuration (11). DFPP also removes plasma, but the amount of supplemental albumin or plasma required is much less than in the standard TPE method because the small molecules removed in the DFPP, mainly albumin, are immediately returned to the patient along with the necessary volume of replenished fluid (13). This provides more selective macromolecule removal and reduces fluid replacement and the need for blood product replacement. DFPP has been reported to have successfully treated multiple autoimmune diseases, such as antibody-mediated rejection and ABO-incompatible kidney transplantation (12). There have been only four cases of DFPP in maternal-fetal hemolysis, two cases of P, and two cases of RhD immunization reported in the literature (from 2003 to 2019). Bek SG and Kamei K showed that DFPP could effectively reduce anti-D antibody validity to alleviate fetal anemia and even avoid fetal intrauterine transfusion (15, 16).

Our case reports demonstrated that not only IgG-D antibody but also IgG-A and B and IgM-A and B antibodies were removed by DFPP, suggesting that DFPP may be favorable for those patients with maternal red cell alloimmunization complicated by RhD and ABO incompatibility hemolysis. Adverse effects during DFPP processing include albumin loss, bleeding tendency, hypotension, allergy, and catheter-related infection (25, 26). In our case, hypoproteinemia during DFPP was observed. Albumin, immunoglobulins, and coagulation factors decreased after DFPP treatment. Replenishment was not necessary when coagulation factors returned to a normal level on the next day and albumin levels stayed above 30 g/L. The level of immunoglobulin dropped over the whole DFPP treatment course, especially IgG; hence, intravenous immunoglobulin infusion was carried out to prevent the potential risk of infections.

Within 72 h of delivery, RhD negative women who have not been previously sensitized should receive a standard dose of RhIg, which binds to and desensitizes RhD antigens that leak into the maternal serum, thereby preventing the production of anti-D in maternal serum (27). The precise mechanism by which anti-D Ig prevents alloimmunization is unknown. Possible mechanisms include the rapid clearance of anti-D-coated D-positive red cells by macrophages and the downregulation of antigen-specific B cells (28–30).

However, RhIg is not indicated for women who have been alloimmunized already (e.g., a titer of ≥32) as RhIg is not able to block or neutralize any previous immunization (31, 32). The two pregnant women we reported had already given birth to newborns with severe RhD incompatible hemolytic anemia and had elevated anti-D antibodies in the maternal circulation, so there was no indication for using RhIg.

## Conclusions

We successfully used DFPP to manage severe early-onset RhD disease before 20 weeks of gestation, saving time for IUT later on. In conclusion, DFPP may be an effective and safe strategy to remove RhD IgG antibody and reduce hemolytic responses in women with RhD-incompatible pregnancies, especially those complicated by ABO-incompatible pregnancies, therefore prolonging pregnancy to receive safe IUT or reducing the need for IUT. However, further clinical trials are needed to verify the results.

## Data Availability

The raw data supporting the conclusions of this article will be made available by the authors, without undue reservation.

## Supplementary material

Supplementary Figure S1 The change of IgM-A and B antibodies during DFPP treatment (case 1). (A) The change of IgM-A titers and the comparison of IgM-A titers pre- and post-DFPP treatment. (B) The change of IgM-B titers and the comparison of IgM-B titers pre- and post-DFPP treatment.

Supplementary Figure S2 The change of IgG-D during DFPP treatment (case 2).

Supplementary Figure S3 The change of IgG-D and MCA-PSV over the whole gestation.
