# Supplementary figures and images for: Case report: Double filtration plasmapheresis (DFPP) for severe rhesus-D alloimmunization in two pregnant patients
Source: Front Pediatr. 2023 Apr 11;11:1147675. doi: 10.3389/fped.2023.1147675 (PMC10127454; doi:10.3389/fped.2023.1147675)

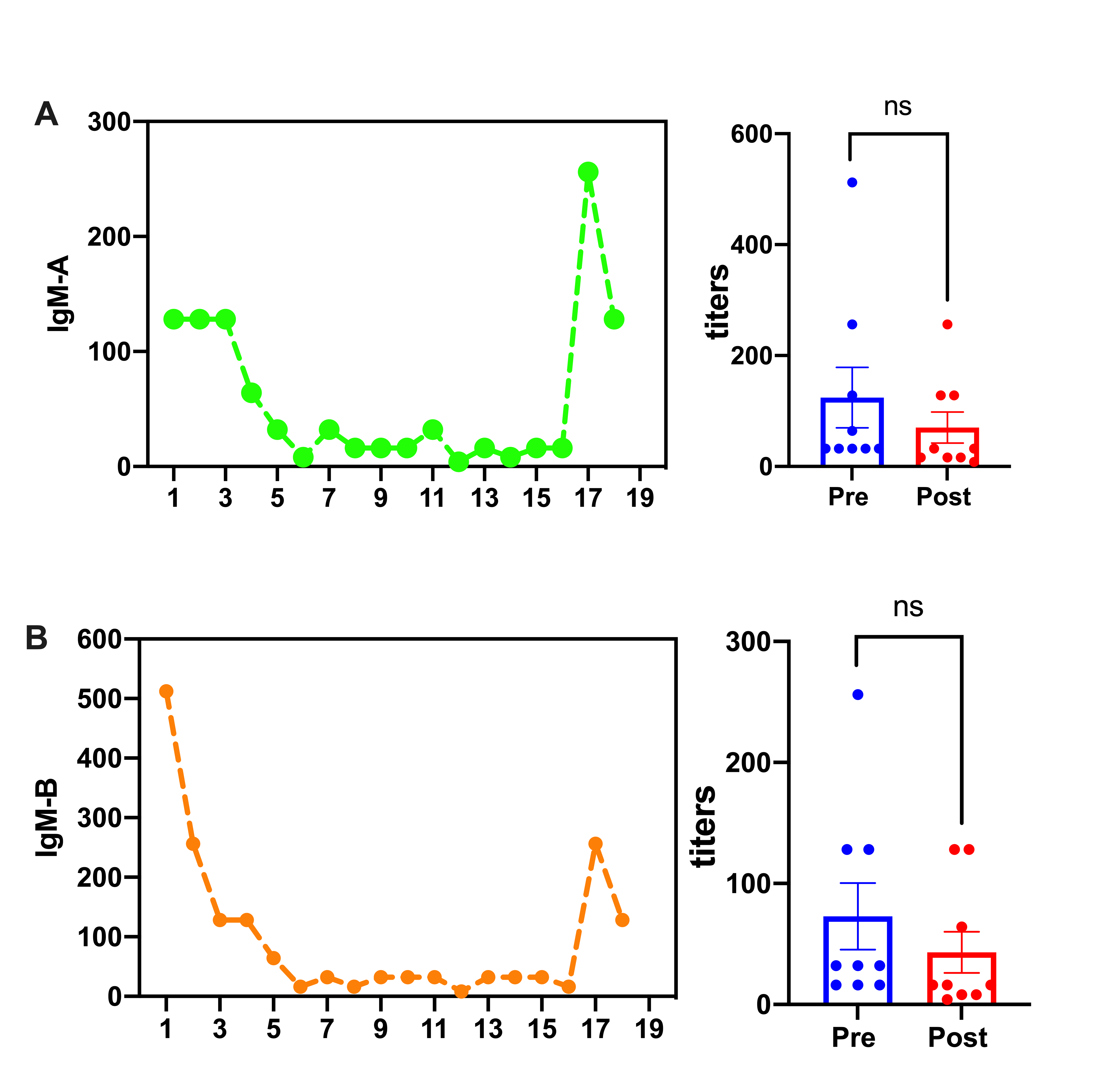

Supplement: Supplementary Figure S1 — The change of IgM-A and B antibodies during DFPP treatment (case 1). (A) The change of IgM-A titers and the comparison of IgM-A titers pre- and post-DFPP treatment. (B) The change of IgM-B titers and the comparison of IgM-B titers pre- and post-DFPP treatment. [file Image1.tiff]

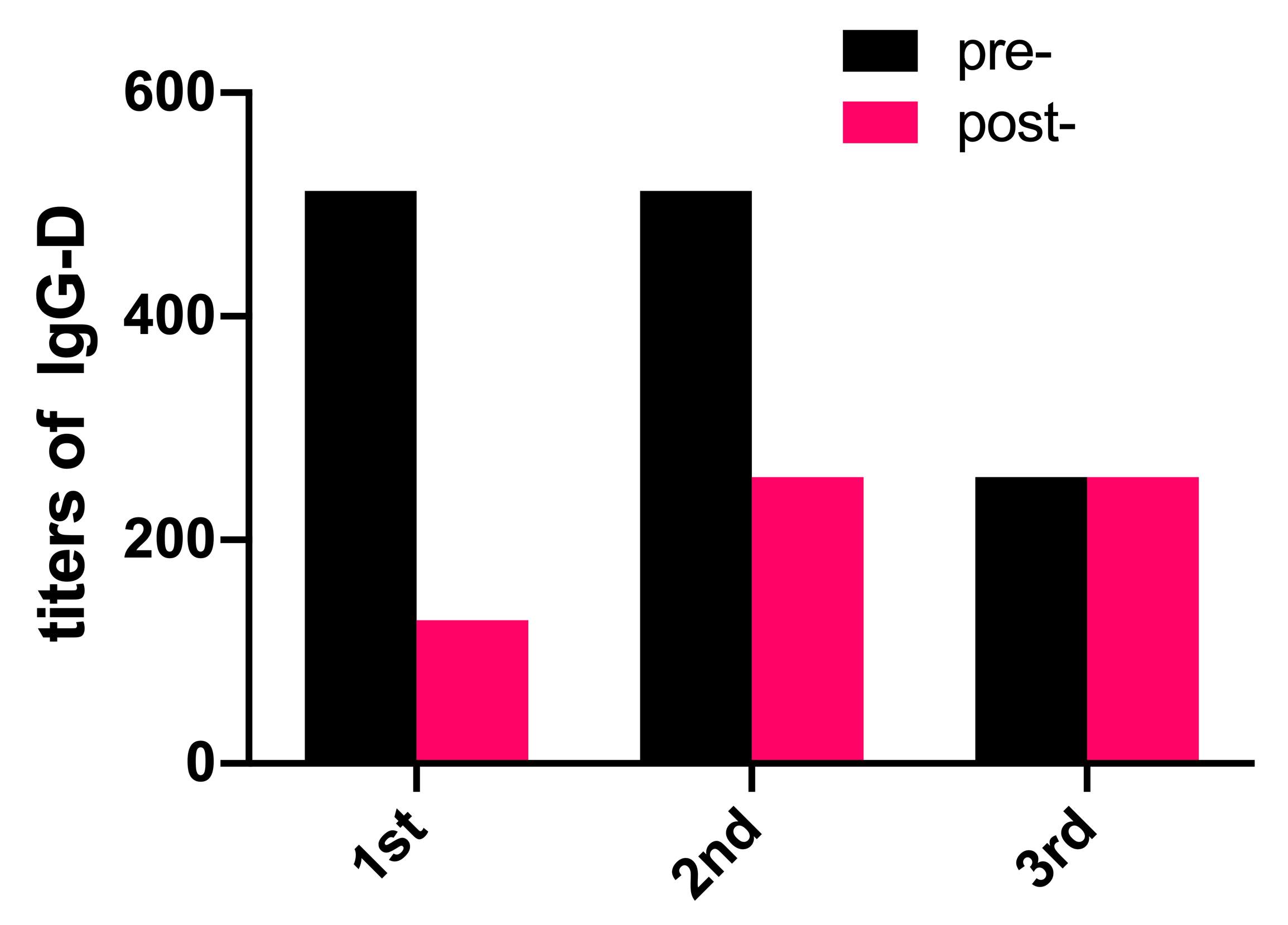

Supplement: Supplementary Figure S2 — The change of IgG-D during DFPP treatment (case 2). [file Image2.tiff]

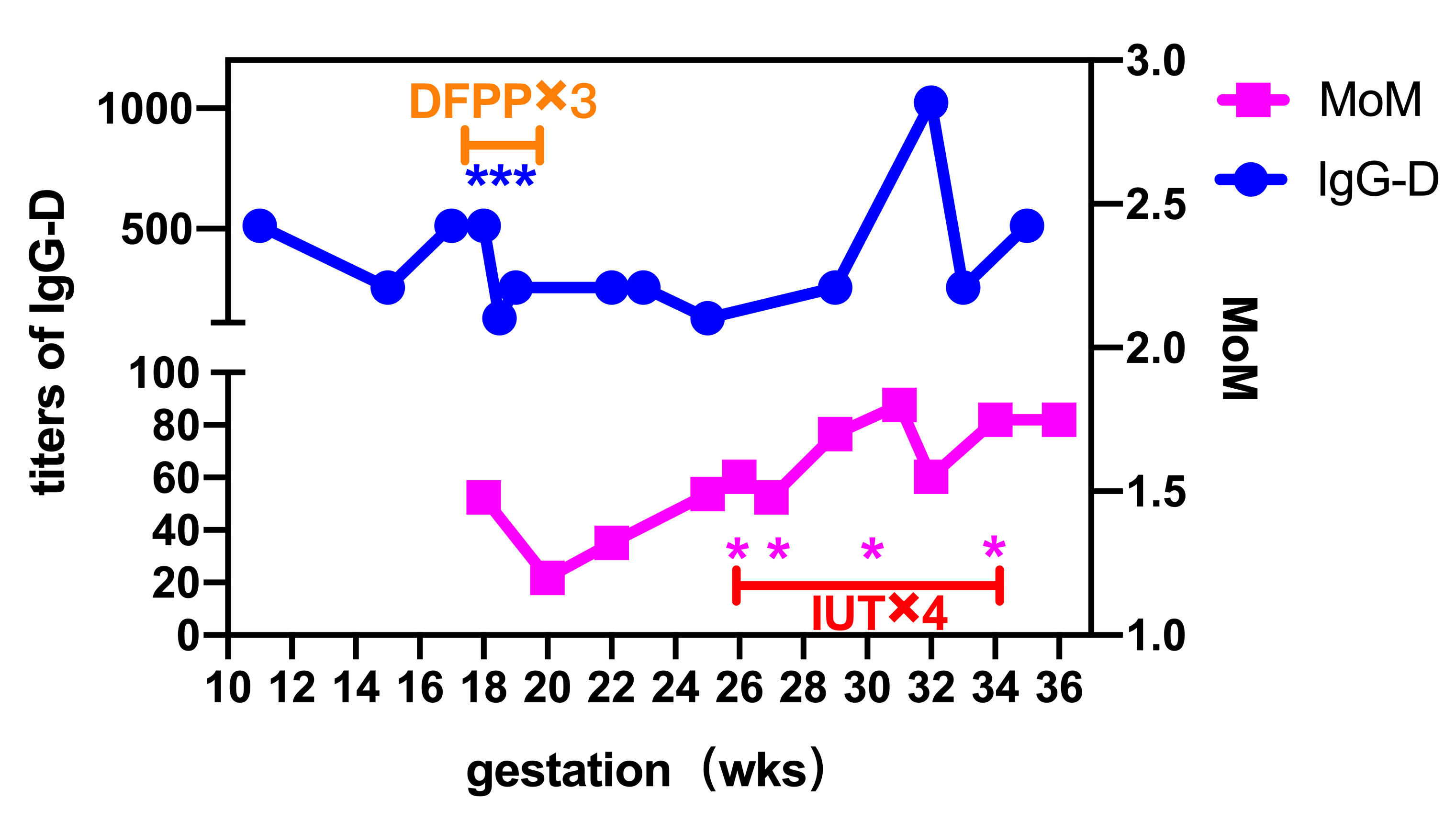

Supplement: Supplementary Figure S3 — The change of IgG-D and MCA-PSV over the whole gestation. [file Image3.tiff]
